# Reply to Burger et al.: Powerless paradigms and the myth of equal fitness

**DOI:** 10.1073/pnas.2631788123

**Published:** 2026-10-23

**Authors:** Geerat J. Vermeij, Rick K. Grosberg, Peter D. Roopnarine

**Affiliations:** ^a^Department of Earth and Planetary Sciences, University of California Davis, Davis, CA 95616; ^b^Department of Ecology and Evolution, University of California Davis, Davis, CA 95616; ^c^ Invertebrate Zoology and Geology, California Academy of Sciences, San Francisco, CA 94118

In their response to the paper by Vermeij et al. (1), Burger et al. (2) reiterate their equal fitness paradigm (EFP) and reject the power-centered view of adaptive evolution (1, 3). The authors claim that, because lineages persist and coexist, individuals in these lineages have equal energetic fitness, which they define as the genetic contribution to the next generation. Besides appearing to equate fitness with persistence—a dubious proposition (4)—they reject the idea that selection, together with individual agency, favors as much per-capita power as circumstances allow, because “species with low-powered life histories would be long extinct.” This is a misunderstanding. Natural selection and agency favor as much power to be acquired and expended regardless of their power status; low-powered organisms and species simply do not have much access to much energy or time, either because of internal inherited constraints of physiology or limits on activity imposed by more powerful organisms. This need not imperil survival or propagation (3), because even the most powerful species cannot effectively eliminate less powerful ones and because the latter help to maintain a workable economic system in the face of change brought about by external events as well as by adaptive evolution.

Burger and colleagues suggest that “If natural selection maximizes power, it must specify which component of the energy budget represented by the EFP equations is optimized.” Leaving aside the question whether evolution optimizes anything, we note that power is a multidimensional quantity, which means that selection and agency have options to maximize any one or more of these dimensions (body mass, number of offspring, force applied by muscles, locomotor speed, and acceleration where applicable, and many more), depending on mode of life (1, 3). We agree that “metabolism cannot be the direct target of selection,” but just because “respired energy is dissipated as heat” does not mean that power acquired and expended to do work is irrelevant to an organism. Work (expressed as energy) takes time, that is, power; and is central to the project of life itself.

The EFP admits brief departures from steady-state demographic equilibria, but at the fine scale of biotic interactions and natural selection, it is these equilibria that are transient. Real communities of coexisting species are never isolated; energy, nutrients, and species flow within and between systems in ways not strongly affected by the activities of organisms, activities that are both the product and cause of agency and natural selection. There may be a fixed amount of energy available to the biosphere at any given time, but this quantity varies and has almost certainly increased in the long run, again in part owing to the adaptive evolution of constituent life. Given this life-induced dynamic, the concept of zero-sum with respect to resources, which is the basis of the EFP, applies only locally and transiently. The history of life does not emerge from steady state constraints.

In their comment and previous papers, Burger and colleagues express the hope for an energy revolution in evolutionary biology. The power-centered perspective, rooted in the lives and interactions of living organisms, offers just what these authors are asking for, without the burden of the concept of fitness, which cannot be applied to fossils and which is unknown in most species. The EFP is founded on faulty assumptions and is not the answer.

## Data Availability

There are no data underlying this work.
